# Regulation of Programmed Death Ligand 1 (PD-L1) Expression in Breast Cancer Cell Lines In Vitro and in Immunodeficient and Humanized Tumor Mice

**DOI:** 10.3390/ijms19020563

**Published:** 2018-02-13

**Authors:** Eva-Maria Rom-Jurek, Nicole Kirchhammer, Peter Ugocsai, Olaf Ortmann, Anja K. Wege, Gero Brockhoff

**Affiliations:** 1 Department of Gynecology and Obstetrics, University Medical Center Regensburg, Landshuter Straße 65, 93053 Regensburg, Germany; nicole.kirchhammer@unibas.ch (N.K.); Peter.Ugoscai@ukr.de (P.U.); oortmann@caritasstjosef.de (O.O.); Anja.Wege@ukr.de (A.K.W.) ; gero.brockhoff@ukr.de (G.B.); 2 Department of Biomedicine, University Hospital Basel, Hebelstrasse, 204031 Basel, Switzerland

**Keywords:** breast cancer cell lines, PD-(L)1, humanized tumor mice, Epirubicin, Paclitaxel

## Abstract

Programmed death ligand 1 (PD-L1) expression is an efficient strategy of tumor cells to escape immunological eradiation. However, only little is known about the factors that affect the cellular expression levels. Here we assessed the PD-L1 expression on different breast cancer cell lines under standard in vitro culture conditions and as a function of Epirubicin or Paclitaxel treatment. Moreover, we evaluated the expression in immunodeficient tumor mice as well as in humanized tumor mice (i.e., in the presence of a human immune system). We found highest PD-L1 levels in JIMT-1 and MDA-MB-231 cells. Epirubicin treatment caused a decrease and Paclitaxel treatment an increased PD-L1 expression in MDA-MB-231 cells. In addition, we identified nuclear PD-L1 in MDA-MB-231 cells. All in vivo transplanted breast cancer cell lines downregulated PD-L1 expression compared to their in vitro counterpart. Neither the gene copy number nor the presence of human immune system in humanized tumor mice had an effect on the PD-L1 content. We demonstrate that the degree of PD-L1 expression amongst breast cancer cell lines varies considerably. In addition, cytotoxic treatments and other extrinsic parameters differentially affect the expression. Hence, further investigations including in vivo evaluations are necessary to understand PD-L1 regulation for advanced breast cancer stratification.

## 1. Introduction

Accumulating evidences suggest a complex tumor-immune-cell interaction in triple-negative and Her2-positive breast cancer (BC) that is caused by a pronounced immunogenicity of these subentities. The extent of an immune response, reflected by the amount of immune cell infiltration, determines BC prognosis [1,2]. The tumor immune cell interactions involve tumor cell elimination, tumor-immune cell equilibrium, but also the escape of tumor cells from the immunological defense, which is—among other mechanisms- achieved by the expression of inhibitory immune checkpoint molecules [3]. Therapeutic strategies designed to stimulate the patient’s inherent immunological tumor defense e.g., by targeting immune checkpoints are considered to enhance conventional treatment regimens. 

A number of immune modulating approaches are currently being tested in clinical trials in order to neutralize the immune escape by tumor cells. A prominent target that contributes to tumor cell evasion is the immune checkpoint programmed death ligand 1 (PD-L1), also referred to as B7-H1 or CD274. If expressed by tumor cells [4], the interaction of PD-L1 with its counterpart, the programmed death receptor 1 (PD-1), leads to T-cell anergy or apoptosis [5,6,7,8]. An aberrant PD-L1 expression has been particularly shown on immunogenic tumor types, such as melanoma [9] and non-small-cell lung cancer (NSCLS) [10]. Ghebeh et al. demonstrated a 50% PD-L1 expression on BC cells as well as on tumor infiltrating lymphocytes (TILs) [11]. Triple-negative breast cancer (TNBC) specimens showed the highest level of PD-L1 expression, followed by Her2 overexpressing subtypes, and lastly the luminal (luminal A and luminal B) entities [11,12,13,14,15]. Further investigations revealed a correlation between PD-L1 expression and increased immune cell infiltration of the tumor [12,13,14,16], which indicates an extensive tumor–immune–cell interaction. PD-L1 expression has repeatedly been associated with a worse outcome [17]. More specifically, enhanced PD-L1 expression goes along with poor prognostic features, such as an increased proliferation index, enlarged tumor size, absence of estrogen receptor (ER) and/or progesterone receptor (PR), Her2 positivity, and a high tumor grade [14]. Paradoxically, Schalper and colleagues described a high PD-L1 mRNA expression associated with increased tumor infiltrating lymphocyte (TIL) rates but an improved recurrence-free survival [18]. In accordance, an increased PD-L1 expression was strongly associated with increased disease-free survival (DFS) but not with overall survival (OS) [16]. Overall, the prognostic and predictive values of PD-L1 expression in BC are uncertain, which might be due to a complex PD-L1/PD-1 interaction and a dynamic regulation of these checkpoint molecules on immune and tumor cells. PD-L1 expression seems to be regulated multifactorially, e.g., by cytokines released by immune cells, and possibly by chemotherapeutic interventions. The cellular distribution of PD-L1 might play a role, as well [19]. The extrinsic and intrinsic mechanisms of PD-L1 regulation in BC requires a complete elucidation as this is crucial for an advanced patient stratification.

Here we analyzed the constitutive PD-L1 expression and the cellular localization in subtype-specific BC cell lines as well as the cellular distribution of ER-positive/Her2-negative, ER/Her2 double-positive, and triple-negative BC cell lines under standardized conditions in vitro and as a function of cytotoxic treatments (i.e., Epirubicin [Epi], Paclitaxel [Ptx]). Moreover, we engrafted triple-negative (MDA-MB-231) and Her2-positive (BT-474, SK-BR-3, and JIMT-1) cell lines in immunocompromised and in humanized tumor mice to assess the potential impact human immune cells on the PD-L1 expression in vivo.

## 2. Results

### 2.1. Constitutive Expression of Programmed Death Ligand 1 (PD-L1) in Different Breast Cancer (BC) Cell Lines

**Figure 1:**
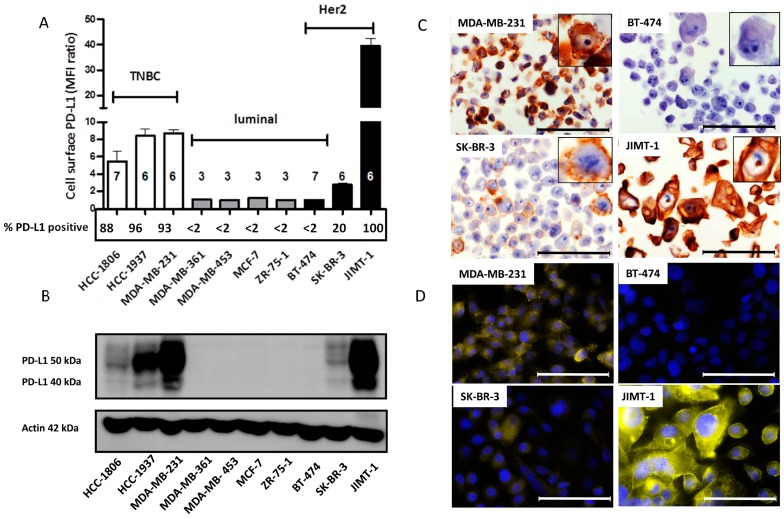
Programmed death ligand 1 (PD-L1) expression on different breast cancer (BC) cell lines. (**A**) Triple-negative breast cancer (TNBC), luminal and Her2 overexpressing BC cell lines were analyzed on basal PD-L1 cell surface expression by flow cytometry (mean fluorescence intensity (MFI) ratio = (MFI_PD-L1_/MFI_isotype_) ± standard error of the mean (SEM)). Numbers of analyzed experiments are indicated above each cell line. Flow cytometric data of % PD-L1 positive cells are presented below each bar for each cell line; (**B**) PD-L1 protein expression was evaluated by western blot analysis. Actin was used as loading control; (**C**) Immunochemical staining of PD-L1 was performed on FFPE embedded BC cell lines. Bars represent 100 µm; (**D**) Immunofluorescence staining of PD-L1 on four representative BC cell lines. Bars represent 100 µm.

The constitutive PD-L1 expression of different BC cell lines under standardized culture conditions was analyzed by flow cytometry, immunochemistry, western blotting, and immunofluorescence (Figure 1). Flow cytometric assessment revealed that the trastuzumab-resistant and Her2-positive JIMT-1 cell line, exhibited the highest PD-L1 cell surface expression and percentage of PD-L1 positive cells followed by TNBC cell lines MDA-MB-231, HCC-1937, and HCC-1806. Hormone receptor-positive (i.e., luminal) BC cell lines, inclusively BT-474, expressed only low levels of cell surface PD-L1 (Figure 1A). In support of the results obtained by flow cytometry, western blotting confirmed that the PD-L1 protein level of each cell line is congruent with its cell surface PD-L1 expression. Again, TNBC cell line subtypes as well as Her2-positive JIMT-1 (and low levels in SK-BR-3) BC cell lines showed the highest PD-L1 expression (Figure 1B). Based on flow cytometry, the TNBC cell lines exhibited a similar cell surface PD-L1 content whereas the total PD-L1 protein concentration detected by western blotting seems to differ to some extent. Immunochemical (Figure 1C) and immunofluorescence (Figure 1D) staining verified the highest PD-L1 expression on JIMT-1 BC cells, with 100% of the cells being PD-L1 positive. In contrast, MDA-MB-231 and SK-BR-3 BC cell lines showed heterogeneous PD-L1 expression with positive and partially PD-L1 negative cells. In agreement with the data assessed by flow cytometry and western blot, BT-474 cells appear as PD-L1-negative.

### 2.2. Assessment of PD-L1 Expression In Vivo Using Tumor Mice (TM) and Humanized Tumor Mice (HTM) Transplanted with Different BC Cell Lines

**Figure 2:**
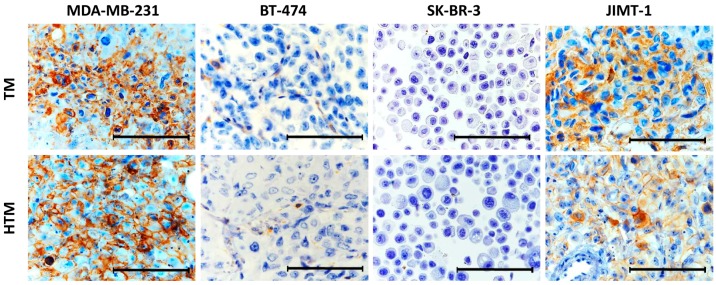
In vivo PD-L1 expression in tumors of tumor mice (TM) and humanized tumor mice (HTM), transplanted with different BC cell lines. Immunohistochemical staining of PD-L1 in tumor samples of TM or HTM transplanted with MDA-MB-231, BT-474, SK-BR-3 or JIMT-1 BC cell lines cotransplanted with or without human hematopoietic stem cells (HSC). Bars represent 100 µm.

PD-L1 expression was immunohistochemically analyzed in an in vivo model based on NOD-*scid IL2Rγ^null^* (NSG) mice that were transplanted with human BC cell lines (MDA-MB-231, BT-474, SK-BR-3, and JIMT-1) with or without a simultaneous intrahepatic transplantation of CD34^+^ hematopoietic stem cells. The transplanted mice developed either solid tumors subcutaneously (MDA-MB-231, JIMT-1), liver-associated tumors (BT-474), or tumor effusions in the peritoneal cavity (SK-BR-3). Moreover, mice transplanted with CD34^+^ cells developed a functional human immune system up to 12 weeks post-transplant. In line with the in vitro data, the highest PD-L1 expression was found in MDA-MB-231 and JIMT-1 BC cell line transplanted animals both in the presence or absence of a human immune system (Figure 2). Interestingly, no PD-L1^+^ tumor cells isolated from the peritoneal effusion were no longer detectable in vivo in tumor mice (TM) nor humanized tumor mice (HTM). However, BT-474, MDA-MB-231, SK-BR-3, and JIMT-1 BC cell line tumors apparently showed diminished PD-L1 expression in vivo compared to in vitro cultured cells. In addition, the expression pattern of PD-L1 in MDA-MB-231 and JIMT-1 TM and HTM tumor tissues was very heterogeneous and not expressed ubiquitously. The human immune system in HTMs did not apparently affect the PD-L1 expression in vivo.

### 2.3. Investigation of PD-L1 Gene Copy Number Variations in Different BC Cell Lines

To assess the potential correlation between PD-L1 protein expression and the *PD-L1* gene copy number TNBC, luminal, and Her2 overexpressing cell lines were analyzed via a PD-L1 specific fluorescent in-situ hybridization (FISH) probe (Table 1). *PD-L1* gene copy numbers were in the normal range in most cell lines (i.e., more or less two signals per nucleus), except HCC-1937 (TNBC), which demonstrated not only an increased *PD-L1* gene copy number but also an increased number of centromere 9 (cen9) signals. MDA-MB-231 and BT-474 BC cells showed a reduced *PD-L1* gene copy number that is also reflected in a *PD-L1*/*cen9* ratio < 1.0. In contrast, MDA-MB-453 and HCC-1806 BC cells displayed only enhanced *cen9* copy numbers (i.e., without increase *PD-L1* gene copy numbers) which indicates some degree of polysomy 9 with a simultaneous loss of chromosomal regions (i.e., *PD-L1*). This finding might indicate instability of chromosome 9. An increased *PD-L1* gene copy number in JIMT-1 BC cells could not be found, although this cell line showed the highest cell surface PD-L1 protein expression (Figure 1). Remarkably, strongly PD-L1-positive MDA-MB-231 cells revealed a loss of the *PD-L1* gene region. Overall, there was no association between the *PD-L1* gene copy number and PD-L1 protein expression indicating that the PD-L1 expression is primarily not determined by the *PD-L1* gene dose. Representative images of FISH probe analyses of MDA-MB-231, BT-474, SK-BR-3, and JIMT-1 cell lines are demonstrated in Supplementary Materials (Figure S1).

### 2.4. Effect of Cytotoxic Treatments on the PD-L1 Expression in MDA-MB-231 BC Cells

**Figure 3:**
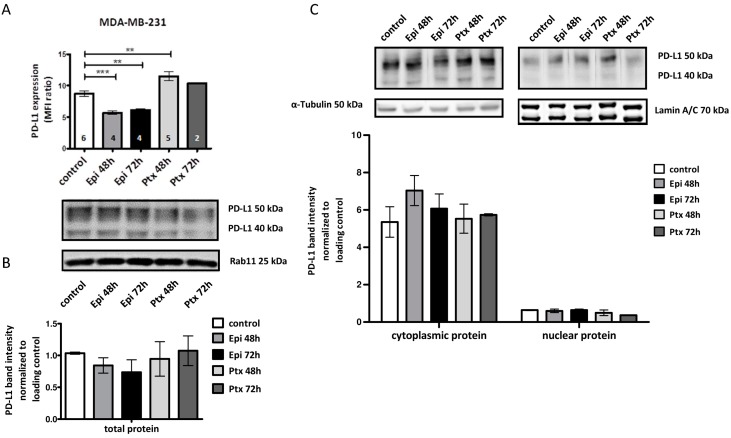
PD-L1 cell surface, total protein, and fractioned protein expression after cytotoxic treatment in MDA-MB-231 BC cell line. (**A**) Flow cytometric measurement of PD-L1 expression on MDA-MB-231 BC cell line after cytotoxic treatment with Epi or Ptx. Data are represented as MFI ratio = (MFI_PD-L1_/MFI_isotype_) ± SEM. Number of repeated measurements are shown in each bar. Significance was calculated using one-way analysis of variance (ANOVA) and Dunnett’s post-hoc test (** = *p* < 0.01; *** = *p* < 0.001); (**B**) Western blot analysis of MDA-MB-231 total protein lysates after cytotoxic treatment. Rab11 was used as loading control. Data are presented as mean ± SEM (*n* = 2); (**C**) Western blot analysis of PD-L1 in cytoplasmic and nuclear protein fraction after cytotoxic treatment. Lamin A/C was used as a loading control for nuclear protein, and α-Tubulin for the cytoplasmic protein fraction. Data are represented as mean ± SEM (*n* = 2). Representative western blots are provided for each dataset.

We assessed the PD-L1 expression in MDA-MB-231 cells upon treatment with Epirubicin (Epi) or Paclitaxel (Ptx) for 48 or 72 h by flow cytometry and western blotting. Interestingly, Epi treatment significantly decreased PD-L1 expression after 48 (*p* < 0.001) and 72 h (*p* < 0.01) (Figure 3A). In contrast, Ptx treatment resulted in enhanced PD-L1 cell surface expression after 48 h (*p* < 0.01) (Figure 3A). The PD-L1 downregulation upon treatment with Epi remained stable for 72 h whereas the Ptx induced PD-L1 upregulation (48 h) dropped back to the basal expression after 72 h. The Epi and Ptx treatment effects on the cellular distribution of PD-L1 (i.e., total protein, vs. cytoplasmic vs. nuclear expression) was further analyzed by western blotting upon total and fractional protein isolation. A tendency towards decreased PD-L1 total protein concentration could be detected after Epi treatment (72 h), however, these differences were not significant (Figure 3B). Fractionated western blotting revealed a most pronounced PD-L1 protein concentration in the cytoplasmic fraction but also nuclear PD-L1 protein in MDA-MB-231 cells was found. In contrast to cell surface PD-L1 expression in MDA-MB-231 BC cell line, PD-L1 cytoplasmic or nuclear protein was not significantly affected by cytotoxic treatment (Figure 3C).

### 2.5. Effect of Cytotoxic Treatments on the PD-L1 Expression in SK-BR-3 BC Cells

**Figure 4:**
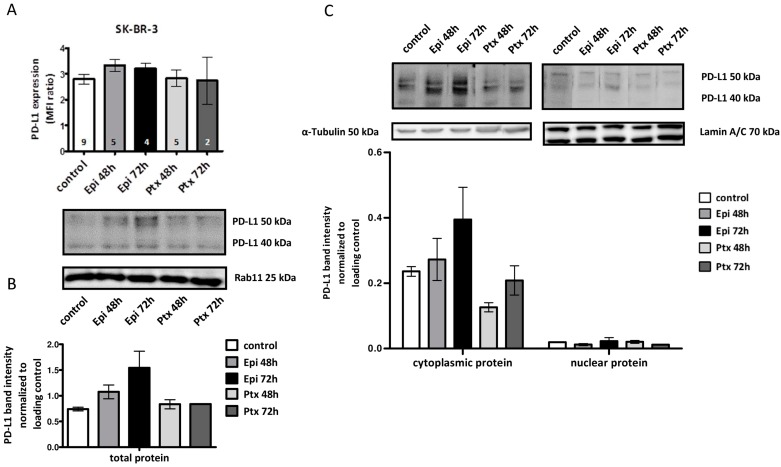
PD-L1 cell surface, total protein, and fractioned protein expression after cytotoxic treatment in SK-BR-3 BC cell line. (**A**) Flow cytometric measurement of PD-L1 expression on SK-BR-3 BC cell line after cytotoxic treatment with Epi and Ptx. Data are represented as MFI ratio = (MFI_PD-L1_/MFI_isotype_) ± SEM. Number of experiments are indicated in each bar; (**B**) Western blot analysis of SK-BR-3 total protein lysates after cytotoxic treatment. Rab11 was used as loading control. Data are presented as mean ± SEM (*n* = 2); (**C**) Western blot analysis of PD-L1 protein expression in cytoplasmic and nuclear fraction after cytotoxic treatment of SK-BR-3 BC cell line. Lamin A/C was used as a loading control for nuclear protein, and α-Tubulin for the cytoplasmic protein fraction. Data are presented as mean ± SEM (*n* = 2). Representative western blots are provided for each dataset.

Cell surface as well as total, cytoplasmic, and nuclear PD-L1 protein expression in Her2-positive SK-BR-3 cells was analyzed upon cytotoxic treatments (Epi or Ptx/48 and 72 h). We found no altered treatment induced PD-L1 cell surface expression nor a redistribution of cell surface located PD-L1 into the nucleus in SK-BR-3 cells (Epi and Ptx after 48 and 72 h) (Figure 4A). However, the total and cytoplasmic PD-L1 protein expression showed a tendency towards enhanced PD-L1 72 h after Epi treatment (Figure 4B,C). As found in MDA-MB-231 BC cells, only low PD-L1 protein levels were detectable in the nuclear fraction without modification by any cytotoxic treatment (Figure 4C). The cell surface located PD-L1 expression was not significantly affected even though a tendency towards an enhanced PD-L1 total protein after Epi treatment was observed.

## 3. Discussion 

To date, (neo-) adjuvant chemotherapy is the only systemic approach for the treatment of triple-negative breast cancer (TNBC) and (besides receptor targeting) an essential element for handling Her2-positive tumors. However, the rate of acquired (multi drug) resistance is high and severe side effects are inevitable. Hence, novel biomarkers that indicate the expected treatment efficacy or those that are useful therapy targets are needed for both tumor entities. So-called “immune checkpoint” molecules might meet those requirements. The programmed death ligand 1 (PD-L1)/ programmed death receptor 1 (PD-1)-axis, for example, has been shown to efficiently enable the tumor to escape from the immunological tumor defense [19,20], however, the regulation of PD-L1 (and PD-1) expression is considered to be highly dynamic and subjected to multifactorial parameters.

In this study, we assessed the constitutive PD-L1 expression as well as the cellular distribution of ER-positive/Her2-negative, ER/Her2-double-positive, and triple-negative breast cancer (BC) cell lines under standardized conditions in vitro and as a function of cytotoxic treatments. Moreover, PD-L1 expression of different BC cell lines was analyzed in an in vivo tumor mouse (TM) and humanized tumor mouse model (HTM). 

In accordance with other studies [11,14,15,21], we report here a high PD-L1 expression in aggressive triple-negative and Her2-positive BC cell lines as well as a very low PD-L1 expression in hormone receptor positive BC cell lines. An enhanced PD-L1 expression in TNBC and Her2 positive tumors in vivo seems to be associated with an enhanced load of neoantigens in those cells. Such a phenotype attracts immune cells and triggers a tumor-immune cell interaction that can finally be inhibited by PD-L1/PD-1 interaction. Nevertheless, even in the same subentity of BC cell lines, a considerable variation of PD-L1 expression can be found—a phenomenon that reflects variations found in patient tumors of the same subentity as well [11]. Moreover, Dill and colleagues demonstrated even a pronounced intratumoral heterogeneity of PD-L1 expression [22]. This observation can obviously be reproduced in vitro since the cell lines used in this study (especially SK-BR-3) likewise showed a heterogeneous and nonubiquitous PD-L1 expression.

Interestingly, the high PD-L1 expression in BC cell lines (i.e., MDA-MB-231 triple-negative and Her2-positive JIMT-1) seems not to be determined by the *PD-L1* gene dose (i.e., an enhanced gene *PD-L1* gene copy number). This finding is substantiated by our recent study on 103 primary TNBC tissue specimens [23] and indicates again a nongenomically predefined PD-L1 expression. The protein expression does not correlate with the *PD-L1* gene copy number and an enhanced *PD-L1* gene/*cen9* ratio was not found. 

Instead, the PD-L1 expression on tumor cells seems to be regulated by external factors and can be induced by cytokines (e.g., IFNγ) released by immune cells, growth factors or hypoxia [21,24]. Therefore, we analyzed the impact of human immune system that matured simultaneously with tumor growth in HTM. However, we did not observe that the PD-L1 expression on BC cells in HTM differs significantly to those tumors that were grown in TM (i.e., in the absence of a human immune system). This finding is probably due to the cotransplantation that ensures the development of tolerance of the human immune system against mouse tissue and allogenic tumor cells. As a consequence, the immune cells seem not to be activated by the tumor cells and thus, no tumor infiltration and IFNγ release occurred. As a result, PD-L1 expression in the primary tumor of HTM is not elevated by the presence of a human immune system.

Nevertheless, SK-BR-3 and JIMT-1 based tumors come with downregulated PD-L1 expression in vivo in TM and HTM compared to their in vitro counterpart. It has been reported that seeding tumor cells at low densities causes an increased nuclear PD-L1 expression, suggesting an association of PD-L1 upregulation as a lack of cell-cell contacts [25]. In contrast, cell line based BC tumors in TM and HTM are tightly packed which might prevent PD-L1 upregulation whereas BC cell lines incubated in vitro (grow at a maximum of 90% confluence) show only loose cell-cell contacts and might therefore express PD-L1 at higher levels. The phenomenon of a differential protein expression as a function of cell density has been reported for a number of markers, as for example the epidermal growth factor receptor expression [26].

In addition, a correlation between proliferation (Ki-67 expression) and PD-L1 protein levels have been described in glioma [27], melanoma and ovarian cancer [28]. Therefore, PD-L1 expression could be reduced in tumors during a non-proliferative phase.

Since (neo-) adjuvant cytotoxic treatment regimens are common, we investigated the effect of an anthracycline and a taxane treatment on PD-L1 expression in vitro. We found PD-L1 downregulated in triple negative MDA-MB-231 cells when exposed to Epirubicin (Epi) (anthracycline) but an upregulation upon exposition to Paclitaxel (Ptx) (taxane). Obviously, respective cytotoxic treatments differentially affect the PD-L1 expression and individual BC cell subtypes might respond in different ways. This observation is in line with other reports found in the literature. For instance, Doxorubicin (an anthracycline) reduced PD-L1 expression in BC cell lines without any alterations of PD-L1 mRNA levels [29] whereas Zhang and colleagues demonstrated an elevated PD-L1 expression in BC cell lines upon Ptx (taxane) treatment [30]. Conceivably, there is an affiliation to the molecular mechanism triggered by the respective cytotoxic substances (spindle inhibitor vs. DNA intercalator). 

Another finding of our study is a time-dependent correlation of the taxane treatment on PD-L1 expression. 48 h Ptx treatment caused an increased PD-L1 expression in MDA-MB-231 cells that dropped back after 72 h to the level of untreated cells. In contrast, the Epi induced PD-L1 suppression remained stable even after 48 and 72 h. These findings demonstrate the dynamic and variable expression range of PD-L1 on tumor cells. With respect to the situation in patient’s tumors it is still uncertain to what extent a modification of PD-L1 expression occurs after chemotherapy treatment and if different PD-L1 levels affect the prognosis of BC patients [14,16,31].

Only few studies demonstrated an emerging role of nuclear PD-L1 expression in circulating tumor cells and its association with short survival rates of colorectal and metastatic prostate cancer patients [25,32]. Therefore, we analyzed a potential cellular redistribution of PD-L1 in selected BC cell lines as a function of cytotoxic treatments. We observed a nuclear presence of PD-L1 in MDA-MB-231 and SK-BR-3 cell line, whereas the cellular localizations and the respective amount of PD-L1 were not affected by cytotoxic treatments. In contrast, Ghebeh and colleagues demonstrated a Doxorubicin-mediated downregulation of PD-L1 surface expression and a redistribution to the nucleus [29]. This apparent discrepancy might be due to a different effect of the used cytotoxic drugs (Epirubicin vs. Doxorubicin) on PD-L1 cellular redistribution. Off note, Ghebeh did not find any altered RNA concentration due to cytotoxic treatment.

Overall, we demonstrate here that the PD-L1 expression and the cellular location is affected by multiple parameters such as 3D cell–cell contact, cytotoxic treatments and others. In addition, we showed that cell lines derived from different BC subentities (i.e., triple-negative, Her2-positive, luminal) respond differently to those factors and therefore represent a dynamically regulated PD-L1 phenotype. Moreover, we observed that even within a given cell line, PD-L1 is heterogeneously expressed. The apparent inconsistency in literature regarding PD-L1 expression might partly be explained by the multifactorial and complex PD-L1 regulation. These ex- and intrinsic factors affecting PD-L1 expression suggests an impact on disease outcome. The evaluation of additional biomarkers that contribute to the regulation of PD-L1 on tumor cells might potentially enhance and specify the prognostic value of PD-L1 expression in BC subentities. Nevertheless, the cell-type-specific regulation of PD-L1 requires further mechanistic exploration.

## 4. Materials and Methods

### 4.1. Breast Cancer Cell Lines

BT-474 (isolated by Lasfargues and Coutinho; ATCC number HTB-20), SK-BR-3 (isolated by Trempe and Old; ATCC number HTB-30), MDA-MB-231 (isolated by Cailleau; ATCC number HTB-26) and JIMT-1 (isolated by Tanner; DSMZ number ACC-589) BC cell lines were used for cell culture experiments and transplantation to generate tumor mice (TM) and humanized tumor mice (HTM). Moreover, HCC-1806 (isolated by Gazdar and Virmani; ATCC number CRL-2335), HCC-1937 (isolated by Gazdar and Virmani; ATCC number CRL-2336), MDA-MB-361 (isolated by Cailleau; ATCC number HTB-27), MDA-MB-453 (isolated by Cailleau; ATCC number HTB-131), MCF-7 (isolated by McGrath; ATCC number HTB-22), and ZR-75-1 (isolated by Engel; ATCC number CRL-1500) BC cell lines were used. BT-474, SK-BR-3, MDA-MB-231, MCF-7, ZR-75-1, MDA-MB-453 were cultured in Dulbecco’s modified essential medium (DMEM) supplemented with 5% fetal calf serum (FCS) and for MDA-MB-361 the medium was supplemented with 10% FCS. JIMT-1, HCC-1806, and HCC-1937 were cultured in Roswell Park Memorial Institute (RPMI) medium 1640 supplemented with 5% FCS.

### 4.2. Generation of NOD-scid IL2Rγ^null^ (NSG) Based TM and HTM

HTM were generated as described previously [33,34,35]. In brief, NOD-*scid IL2Rγ*^null^ (NSG) mice were obtained from Jackson Laboratories and bred and kept in a specialized pathogen-free facility at the University of Regensburg. HTM were generated by irradiating (1 Gy) newborn animals within the first 48 h after delivery. Three hours later the mice were intrahepatically transplanted with 2.5 × 10^5^ human CD34^+^ cells isolated from umbilical cord blood together with a subcutaneous or intra-hepatic injection of 3 × 10^6^ BT-474, SK-BR-3, MDA-MB-231, and JIMT-1 tumor cells. In addition, cell lines were transplanted subcutaneously or intrahepatic into newborn irradiated animals without human CD34^+^ cells to generate tumor mice (TM).

### 4.3. Ethic Statements

The local veterinary authorities of the district government of Lower Franconia and Upper Palatinate (Bavarian region) approved all animal work based on national regulations of the German animal protection act and the European guidelines (permission no. 54-2532.1-27/11 (3 November 2011), 54-2532.1-44/13 (5 November 2014), 55.2 DMS-2532-2-260 (15 July 2016)).

Cord blood samples were taken based on the approval given by the Ethics Committee of the University of Regensburg (permission no. 11-101-0287 (23 November 2011), 15-101-0057 (22 April 2015)). All patients included in the study provided written informed consent.

### 4.4. Flow Cytometric Assessment of Programmed Death Ligand 1 (PD-L1)

The basal PD-L1 (CD274) expression and the expression after cytotoxic treatment of the different BC cell lines was determined by flow cytometry using a FACSCanto-II flow cytometer run by the Diva software Ver. 7.0 (BD Biosciences, San Jose, CA, USA). The cell lines were seeded in T75 flasks at a concentration of 3 × 10^5^ cells/sample and were grown until they reached a confluence of approximately 70%. Cells were treated with 20 ng/mL Epirubicin (Epi) (Teva, Ulm, Germany) or 5 ng/mL Paclitaxel (Ptx) (ACCORD Healthcare GmbH, Freilassing, Germany) for 48 and 72 h, respectively. Appropriate sublethal concentration of Epi and Ptx was evaluated based on titrations within the range of 0–100 ng/mL. Untreated cells served as control samples to assess the basal PD-L1 expression. The cells were washed, detached from culture flask, counted, and stained with phycoerythrin (PE)-conjugated anti-CD274 antibody (clone 29E.2A3, BioLegend, San Diego, CA, USA). The appropriate mouse immunoglobulin IgG2b,κ PE (clone MPC-11, BioLegend, San Diego, CA, USA) was used for each condition (basal PD-L1 level or the PD-L1 expression after chemotherapeutic treatment) as isotype control. 

### 4.5. Immunohistochemistry

Cell suspensions and tissue samples (spleen, liver, and tumor) were fixed in 5% formalin, embedded in paraffin (FFPE), and cut into 1.5 µm slices. Specimens were deparaffinized followed by rehydration in a descending ethanol gradient (100%, 80%, 70%). Antigen retrieval was done with Tris/EDTA buffer (pH 9) at 121 °C for 5 min using the decloaking chamber. The slides were cooled down for 10 min in a water bath. After blocking endogenous peroxidase (Dako, Hamburg, Germany), the slides were washed in TBS-T for 5 min and the primary antibody PD-L1 (clone E1L3N, Cell Signaling, Danvers, MA, USA) was applied in appropriate dilution (1:200) for 1 h. Afterwards, the sections were washed in TBS-T and the secondary antibody was applied for 30 min at room temperature. The sections were washed again and incubated for 10 min with DAB plus substrate-chromogen solution (Dako, Santa Clara, CA, USA). The specimens were rinsed with distilled water and counterstained with hematoxylin for 2 min. Then the sections were rinsed in tap water (5 min), distilled water (1 min) and, dehydrated in ascending ethanol gradient (70%, 80%, 100%). After 2 × 5 min cleaning steps the specimens were cover-slipped with xylene containing mounting medium.

### 4.6. Immunofluorescence Staining

MDA-MB-231, BT-474, SK-BR-3 and JIMT-1 BC cell lines were cultured in chamber slides (Merck-Millipore, Darmstadt, Germany) up to a confluence of 90%. Immunofluorescence staining was performed according to manufactures protocol of the primary anti-PD-L1 antibody (Cell Signaling). In brief, the medium was removed and the cells were washed with PBS, PBS was discarded, ice-cold 100% methanol was added and incubated for 10 min at −20 °C. Then the slides were rinsed with PBS for 5 min and incubated with blocking buffer for 60 min. The blocking buffer was aspirated afterwards and the primary anti-PD-L1 antibody (1:100) (D8T4X, Cell Signaling, Danvers, MA, USA) or the isotype control (1:833) (DA1E, IgG XP^®^ Isotype Control, Cell Signaling, Danvers, MA, USA) were applied in appropriate dilution for overnight incubation at 4 °C. After a 3 × 5 min washing step with PBS, the slides were incubated for 1 h at room temperature in the dark with goat anti-rabbit IgG AlexaFluor 532 secondary antibody (1:500) (Thermo Fisher Scientific, Bremen, Germany). The slides were washed again (3 × 5 min) with PBS and were cover-slipped with VECTASHIELD Antifade Mounting Medium with DAPI (VECTOR Laboratories Inc., Burlingame, CA, USA).

### 4.7. Western Blotting of Total and Fractionized Proteins

BC cell lines were treated in vitro with 20 ng/mL Epi (Teva, Ulm, Germany) or 5 ng/mL Ptx (ACCORD healthcare GmbH, Freilassing, Germany) for 48 and 72 h. Untreated cells were used as control samples. In order to assess the basal PD-L1 content, BC cell lines were lysed for total protein analysis in cell-lysis buffer (Cell Signaling, Danvers, MA, USA) supplemented with Halt™ Protease and Phosphatase Inhibitor Cocktail (Thermo Fisher Scientific, Bremen, Germany). Cytoplasmic and nuclear proteins were extracted using NE-PER™ Nuclear and Cytoplasmic Extraction kit according to manufactures protocol (Thermo Fisher Scientific, Bremen, Germany). Twenty-five µg protein per lane (total protein) and 17 µg protein per lane (nuclear and cytoplasmic) were separated in 7.5% SDS-PAGE under reducing conditions (mercaptoethanol), and plotted onto Polyvinylidene Difluoride (PVDF) membranes. Membranes were blocked with 5% low-fat milk for 2 h and then incubated overnight at 4 °C with anti-PD-L1 antibody (1:1000; clone E1L3N, Cell Signaling, Danvers, MA, USA) and anti-β-actin (1:15,000; clone AC-15, Sigma-Aldrich, Taufkirchen, Germany), or anti-Rab11 (Cell Signaling, Danvers, MA, USA) as loading control for total protein. The monoclonal E1L3N anti-PD-L1 antibody is known to visualize a glycosylated (~50 kDa) and a non-glycosylated (~40 kDa) variant of PD-L1. For cytoplasmic fraction anti-α-Tubulin (1:1000; Cell Signaling, Danvers, MA, USA) and for nuclear fraction anti-lamin A/C (1:1000; Cell Signaling, Danvers, MA, USA) were used as loading control and in order to exclude potential cross-contaminations of the fractions. As protein size standard, the precision plus protein Western C marker (Bio-Rad, Munich, Germany) was used. Finally, membranes were hybridized with horseradish peroxidase (HRP)-conjugated goat-anti rabbit IgG (1:2000; Cell Signaling, Danvers, MA, USA) and HRP-conjugated horse anti-mouse IgG (1:2000; Cell Signaling, Danvers, MA, USA). The blots were visualized using the chemiluminescent western blotting detection system (GE Healthcare, Amersham, UK) and analyzed by ImageQuant LAS 4000 mini imager (GE Healthcare, Buckinghamshire, UK).

### 4.8. Fluorescence In-Situ Hybridization

Tissue microarrays (TMA) were constructed from FFPE tumor cell pellets. As previously described in detail [36], TMA sections were applied on charged slides (SuperFrost Plus; Menzel GmbH, Braunschweig, Germany) and Fluorescence in-situ hybridization (FISH) was performed using the directly labeled dual color probe CD274, PDCD1LG1/CEN9 (ZytoVision GmbH., Bremerhaven, Germany). The PD-L1 specific probes were labeled with ZyGreen™ (absorption 530 nm, emission 528 nm) and the centromeric probes were labeled with ZyOrange™ (absorption 547 nm, emission 528 nm). The *centromere 9* (*cen9*) region was taken as a surrogate for the number of chromosome 9. The dual marker probe enables to interpret a potential gain of PDCD1LG1 either as a *PD-L1* gene amplification or to chromosome 9 polysomy. In brief, 3–4 µm thick deparaffinized tissue sections were pretreated in 98 °C 0.01 M Na-Citrate buffer for 30 min and incubated with pepsin (ZytoVision GmbH, Bremerhaven, Germany) for 5 min at 37 °C. The sections were rinsed with distilled water and dehydrated in ascending ethanol gradient (70%, 80%, and 100%). Hereafter, 10 µL of the original probe were pipetted on each specimen. The slides were then covered by a coverslip and rubber cement (fixogum™) and after a denaturation step of 5 min at 73 °C the slides were incubated over night at 37 °C. Finally, the coverslip was removed, the samples were washed, and nuclear counter stained with 4′,6-diamidino-2-phenylindole (DAPI). Hybridization spots were then visualized by AxioImager-Z1 microscope (Zeiss, Oberkochen, Germany) and 25 hybridization spots of nonoverlapping nuclei were counted manually on a cell-by-cell basis.

### 4.9. Statistical Analysis

Results are shown as mean ± SEM. For groupwise comparison, a one-way analysis of variance (ANOVA) with Dunnett’s post-hoc test was performed by using the GraphPad Prism 5 software (GraphPad Software, San Diego, CA, USA). *p* values of ≤ 0.05 were considered as significant differences.

## Figures and Tables

**Table 1:** Assessment of Programmed Death Ligand 1 (*PD-L1*) gene copy number in breast cancer (BC) cell lines. *PD-L1* and *cen9* gene signals derived from 25 cells (and calculated as signal per one cell) as well as PD-L1/cen9 ratio are presented. TNBC: triple-negative breast cancer.

Subentities	TNBC			Luminal				Her2		
Breast Cancer Cell Line	HCC-1806	HCC-1937	MDA-MB-231	MDA-MB-361	MDA-MB-453	MCF-7	ZR-75-1	BT-474	SK-BR-3	JIMT-1
*PD-L1*/25 cells	45	89	25	39	44	45	58	25	56	60
(Ø signals/cell)	(1.8)	(3.6)	(1.0)	(1.6)	(1.8)	(1.8)	(2.3)	(1.0)	(2.2)	(2.4)
*cen9*/25 cells	77	85	64	49	84	46	57	75	59	58
(Ø signal/cell)	(3.1)	(3.4)	(2.6)	(2.0)	(3.4)	(1.8)	(2.3)	(3.0)	(2.4)	(2.3)
*PD-L1*/*cen9* ratio	0.58	1.05	0.39	0.8	0.52	0.98	1.02	0.33	0.95	1.03

## Supplementary Materials

Supplementary materials can be found at http://www.mdpi.com/1422-0067/19/2/563/s1.

## Abbreviations

BC	Breast cancer
Epi	Epirubicin
Ptx	Paclitaxel
TM	Tumor mouse
HTM	Humanized tumor mouse
