# Supplementary material for: Regulation of Programmed Death Ligand 1 (PD-L1) Expression in Breast Cancer Cell Lines In Vitro and in Immunodeficient and Humanized Tumor Mice
Source: Int J Mol Sci. 2018 Feb 13;19(2):563. doi: 10.3390/ijms19020563 (PMC5855785; doi:10.3390/ijms19020563)
Supplement: Supplementary file 1 [file ijms-19-00563-s001.pdf]

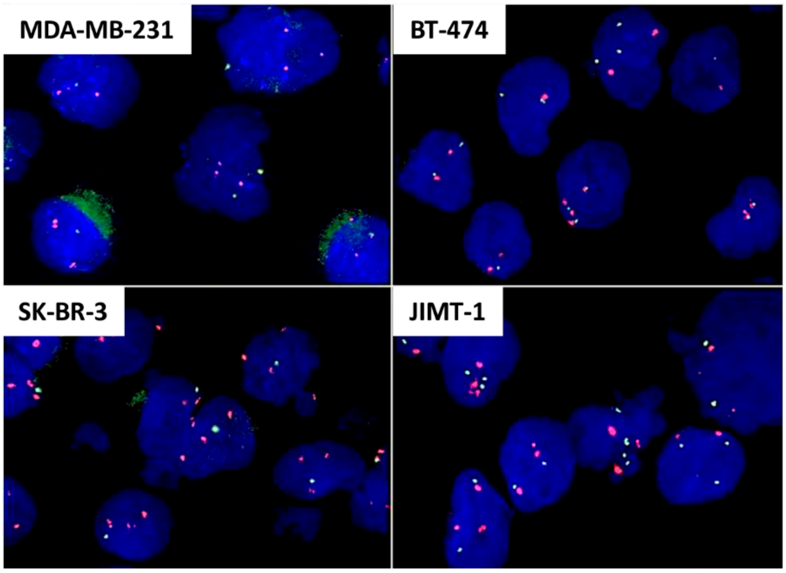

Figure S1. Representative image of FISH analysis in different BC cell lines. MDA-MB-231, BT-474, SK-BR3, and JIMT-1 cell lines were analyzed for PD-L1 (green) and Cen9 (red) gene copy numbers.
